# A Real-Time PCR-Based Semi-Quantitative Breakpoint to Aid in Molecular Identification of Urinary Tract Infections

**DOI:** 10.1371/journal.pone.0061439

**Published:** 2013-04-23

**Authors:** Wendy L. J. Hansen, Christina F. M. van der Donk, Cathrien A. Bruggeman, Ellen E. Stobberingh, Petra F. G. Wolffs

**Affiliations:** Department of Medical Microbiology, Care And Public Health Research Institute (CAPHRI), Maastricht University Medical Center, Maastricht, The Netherlands; The University of Hong Kong, Hong Kong

## Abstract

This study presents a novel approach to aid in diagnosis of urinary tract infections (UTIs). A real-time PCR assay was used to screen for culture-positive urinary specimens and to identify the causative uropathogen. Semi-quantitative breakpoints were used to screen for significant bacteriuria (presence of ≥10^5^ CFU/ml of uropathogens) or low-level bacteriuria (containing between 10^3^ and 10^4^ CFU/ml of uropathogens). The 16S rDNA-based assay could identify the most prevalent uropathogens using probes for *Escherichia coli*, *Pseudomonas* species, *Pseudomonas aeruginosa*, *Staphylococcus* species, *Staphylococcus aureus*, *Enterococcus* species and *Streptococcus* species. 330 urinary specimens were analysed and results were compared with conventional urine culture. Using a PCR Ct value of 25 as semi-quantitative breakpoint for significant bacteriuria resulted in a sensitivity and specificity of 97% and 80%, respectively. In 78% of the samples with monomicrobial infections the assay contained probes to detect the bacteria present in the urine specimens and 99% of these uropathogens was correctly identified. Concluding, this proof-of-concept approach demonstrates that the assay can distinguish bacteriuria from no bacteriuria as well as detect the involved uropathogen within 4 hours after sampling, allowing adequate therapy decisions within the same day as well as drastically reduce consequent urine culturing.

## Introduction

Molecular techniques are becoming an integral part of a diagnostic microbiological laboratory. Many microbiological laboratories performing real-time PCR already offer a broad panel of bacterial and viral targets. Though, for some infections conventional testing procedures are still being used. Urinary tract infections (UTIs) comprise one of the largest classes of infections occurring both in hospital and in community [1], [2], [3]. The diagnosis of UTIs is based on semi-quantitative urine culture, used as reference standard, which provides both quantification as well as identification of the uropathogen. Quantification of uropathogens is essential as different bacterial loads (>10^3^ or 10^5^ CFU/ml) are used in combination with clinical symptoms to identify UTIs in different population groups [4]. Obtaining results from a semi-quantitative culture requires at least 18–24 h. In addition, previous studies have shown that depending on the population, up to 80% of urine cultures are negative, underlining the need for new, less time-consuming and labour-intensive methods [3], [5], [6], [7].

To increase the rapidity of identification of UTIs, rapid urinalysis tools are available and include for example testing for nitrite and leukocytes, and microscopic sediment analysis for bacteria and white blood cells. These screening tools are fast, but often lack sensitivity [8], [9]. Reports associated the nitrite test with a sensitivity and specificity of 45–60% and 85–98%, respectively, leukocyte-esterase testing has been shown to have sensitivity and specificity rates of 48–86% and 17–93%, respectively [8]. The rapidity and ease of use of urine dipsticks are particularly preferred by general practitioners, whereas microbiological laboratories demand solutions designed for high-throughput analysis. An example of an automated urinalysis instrument is the Sysmex urine fluorescence flow cytometer (Sysmex UF-1000i). This instrument uses an algorithm which combines the quantitative detection of bacteria and white blood cells to determine if infection is present. Although the Sysmex system, in contrast to the dipsticks, is able to adjust settings to cover either bacterial loads ≥10^3^ or 10^5^ CFU/ml, this system, as well as the dipstick testing, shows limitations in clinical sensitivity and does not provide an identification of the uropathogen involved. Especially in a hospital setting, where often a larger variation in UTI aetiology is seen as compared to at the general practitioners, uropathogen identification might prove essential in rapid adequate treatment.

The aim of this study was to develop a new molecular approach accounting for both rapid semi-quantification of the bacterial load in urine as well as the identification of the uropathogen. This UTI screening assay was based on the earlier described multi-probe 16S rDNA-based real-time PCR assay using species- and genus-specific probes [10]. The most promising new feature evaluated in the current study was the semi-quantitative breakpoint to distinguish between positive and negative UTI samples. The discriminatory breakpoint was based on the scattering in cycle threshold (Ct) values of the universal 16S rDNA probe. In this study, we defined significant bacteriuria as a uropathogen load ≥10^5^ CFU/ml. Bacterial loads below 10^5^ CFU/ml were considered to be no UTI. In specific patient groups it could be indicated to include a subgroup of low-level bacteriuria as a load between 10^3^–10^4^ CFU/ml. The detection of this group was also investigated in the study. The other purpose of the UTI screening tool was to offer a rapid indication about the causative agent. The pathogens selected as target in the real-time PCR assay were *Escherichia coli*, *Pseudomonas* species, *Pseudomonas aeruginosa*, *Staphylococcus* species, *Staphylococcus aureus*, *Enterococcus* species, and *Streptococcus* species.

## Methods

### Ethics Statement

All data in this study were analyzed anonymously, and the samples were considered to be medical waste materials. Therefore, no consent from the patients was required and the ethics committee did not have to be approached. This is in agreement with the code for proper use of human tissue as formulated by the Dutch Federation of Medical Scientific Societies and the policy of the Medical Ethics Committee of the Maastricht University Medical Center.

### Clinical Samples

We included 330 randomly selected routine clinical urine samples submitted to the Medical Microbiology Laboratory of the Maastricht University Medical Center (MUMC, the Netherlands) for urine culture analysis. The samples were collected from symptomatic and asymptomatic in- and outpatients. The median age of the patients was 64 years old (range 0–92 years, 10% ≤20 years, 21% 21–50 years, 69% >50 years old), 56% of the patients were female. Conventional processing of the urine specimens consisted of dipstick testing for nitrite and/or further pathogen identification by Gram-staining and biochemical testing. Samples were cultured using standard microbiological methods. Identification and antibiotic susceptibility testing was performed with the Phoenix system (BD). All samples were used according to the code for proper use of human tissue as formulated by the Dutch Federation of Medical Scientific Societies.

### Processing of Urine Samples

1 ml of urine sample was centrifuged for 5 min (13.400×g). The supernatant was removed and the remaining pellet was washed with 900 µl phosphate buffered saline (PBS) and centrifuged for 5 min (13.400×g). Again, the supernatant was removed and the pellet was incubated in a lysozyme-lysostaphyn (0.1 mg–0.01 mg) mixture for 15 min at 37°C. After this pre-treatment, DNA isolation was performed with the QIAamp DNA Mini Kit according to the manufacturer’s instructions (Qiagen GmbH, Hilden, Germany). Finally, DNA was eluted in 100 µl of nuclease-free H_2_O. All samples were processed including isolation and amplification controls.

### Multiprobe Assay

The primers and the universal bacterial TaqMan probe have been described previously [11]. The probes for *Pseudomonas* spp., *P. aeruginosa*, *E. coli*, *Staphylococcus* spp., *S. aureus*, *Enterococcus* spp., *Streptococcus* spp. were previously designed and described [10]. Each test contained 5 µl purified sample and 20 µl reaction mixture. The reaction mixture contained 12.5 µl of Taqman Environmental Master Mix 2.0 (Applied Biosystems, Foster City, CA, USA), 0.9 µM of forward primer, 0.6 µM of reverse primer, and 0.2 µM of each probe. There were four separate reactions: I) universal probe, *Pseudomonas* spp. probe and *E. coli* probe, II) *Pseudomonas aeruginosa* probe, III) *Staphylococcus* spp. probe, the *S. aureus* probe and the *Enterococcus* spp. probe, IV) *Streptococcus* spp. probe. Reactions were performed on the ABI PRISM® 7900 real-time PCR System (Applied Biosystems, Foster City, CA, USA) and optimal thermal cycling conditions were as follows: 10 min at 50°C, initial denaturation at 95°C for 15 min, 42 cycles of denaturation for 15 s at 95°C and annealing at 60°C for 1 min. Cycle threshold (Ct), the cycle number at which amplicon fluorescence exceeded the preset detection threshold, was recorded for all samples. The threshold for the Ct analysis was manually adjusted to 0.1, together with the baseline start and end (cycle):6–15.

### Statistical Analysis

The positive predictive value (PPV), negative predictive value (NPV), sensitivity and specificity were calculated for the real-time PCR using the presence of ≥10^5^ CFU/ml in culture as gold standard. For the statistical analysis, data were analyzed using SPSS software, version PASW Statistics 18 (SPSS Inc, Chicago IL, USA). Receiver Operator Characteristics (ROC) curve analysis was performed to determine a real-time PCR-based semi-quantitative breakpoint. The Mann-Whitney U Test and Kruskal-Wallis Test were used to enable correlation of the PCR Ct values with the bacterial load determined in culture. A p-value of <0.05 was considered significant.

## Results

### Population and Assay Characteristics

The collection of urine specimens consisted of 330 samples, of which 279 samples were analyzed retrospectively using the multiprobe assay. Six samples were excluded because of insufficient sample volume, 43 urine specimens were determined as skin/mixed flora and considered contaminants and 2 samples were excluded because of a fungal infection. In our test collection, the prevalence of a positive urine culture was 38%. The complete assay including DNA extraction and real-time PCR could be performed within 4 hours.

### A Semi-quantitative Breakpoint for the Detection of Significant Bacteriuria

**Figure 1:**
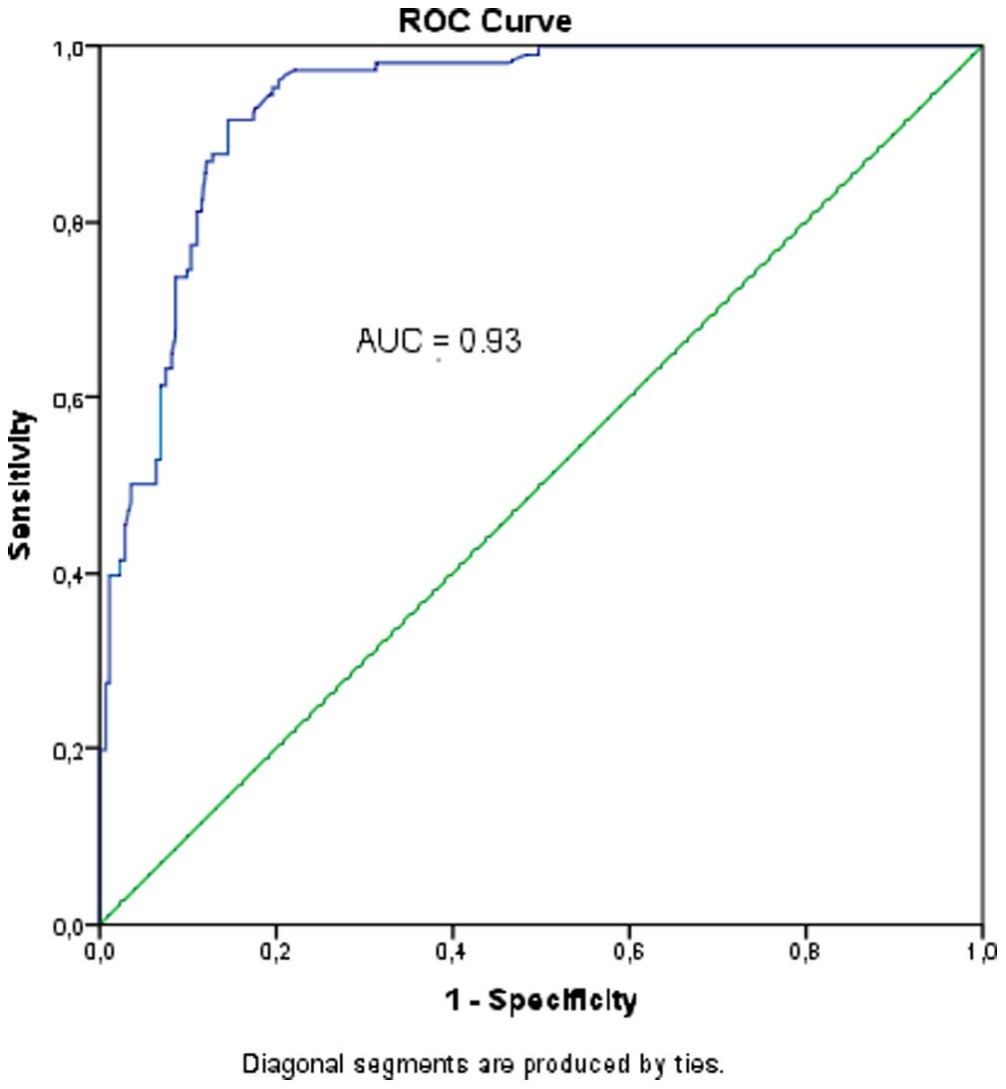
Receiver Operator Characteristic (ROC) decision plot. ROC curve analysis obtained by using the real-time PCR Ct values (universal probe) versus urine culture results (Cut-off value of ≥10^5^ CFU/ml).

**Figure 2:**
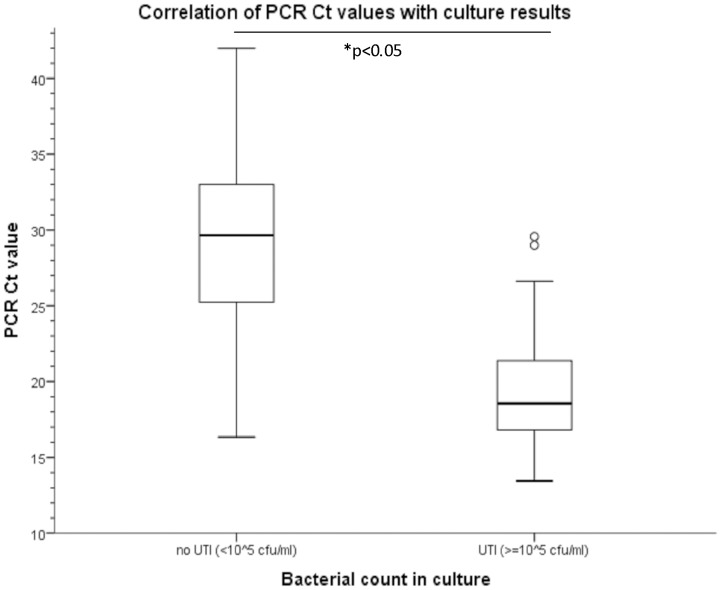
Correlation of PCR Ct values with culture results. Based on culture, samples were categorized into two groups: UTI (≥10^5^ CFU/ml) and no UTI (<10^5^ CFU/ml). The universal 16S rDNA probe, of which a Ct value of 25 was set as breakpoint, was used to distinguish between positive and negative samples (line in dots). * p<0.05 (non-parametric Mann-Whitney Test).

Within our University Medical Center, urine samples (in conjunction with the matching clinical symptoms) are categorized into two groups: UTI if bacteria are present at a concentration equal or higher than 10^5^ CFU/ml and no UTI if the concentration of the bacteria is less than 10^5^ CFU/ml. In this study, we also wanted to determine a semi-quantitative real-time PCR-based cut-off value to distinguish between UTI and no UTI. Therefore, the universal 16S rDNA probe was used as target, and Ct values were related to the cut-off value applied in culture. To evaluate the accuracy and the discriminating power of our diagnostic test, a ROC curve was made (Figure 1). The real-time PCR assay showed a good accuracy (AUC = 0.93). Following, the threshold used to discriminate between positive and negative urine samples was set to a Ct value of 25. At this threshold, 103 samples with UTI were found to be PCR positive and 3 samples negative, whereas 138 samples without UTI were PCR negative and 35 samples were found to be false PCR positive. This resulted in a significant difference in Ct value between the UTI and no UTI group (Figure 2). Using this cut-off value, a sensitivity and specificity of 97% and 80% respectively, could be reached. The positive and negative predictive values were 75% and 98%, respectively (Table 1).

**Table 1:** Comparison of performance characteristics of the diagnostic test (real-time PCR assay, universal probe) using different PCR Ct cut-off values (25–30).

Cut-off value	Sensitivity	Specificity	PPV	NPV
**24**	92	83	77	95
**25**	97	80	75	98
**26**	97	73	69	98
**27**	98	65	63	98
**28**	98	59	59	98
**29**	98	54	57	98
**30**	100	48	54	100

Ct 25 was used as definitive cut-off value.

As shown in Figure 2, two culture-positive samples were false-negative in our diagnostic PCR assay (Ct value of 29.56 and 26.62). An internal amplification control showed that the late signals were not due to inhibition (data not shown). The first sample contained *Enterococcus faecalis* (10^5^ CFU/ml), and the second *Enterobacter cloacae* (>10^5^ CFU/ml). Furthermore, we observed thirty-five false-positive results with PCR, of which in five cases the culture report mentioned the presence of a specific pathogen between 10^4^ and 10^5^ CFU/ml. In the remaining thirty cases no pathogens were found in culture. Adjusting the cut-off value to Ct 30 could resolve the two false-negative results. However, then the amount of false-positive isolates would increase to 90 cases. In Table 1, a summary is given of the performance characteristics of the PCR assay using alternative cut-off values.

**Figure 3:**
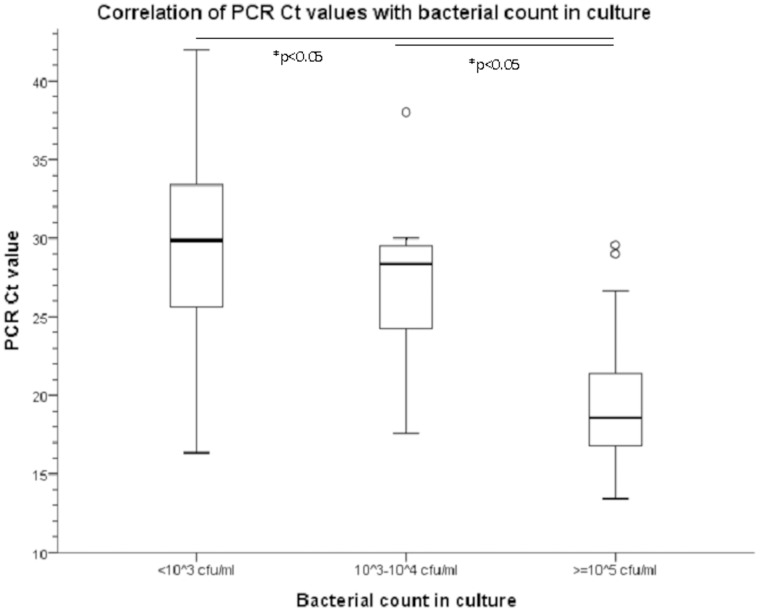
Boxplot showing the correlation of PCR Ct values with bacterial load determined in culture. Culture results were grouped into three categories: <10^3^ CFU/ml, 10^3^–10^4^ CFU/ml and ≥10^5^ CFU/ml. Statistical significance was determined performing Kruskal-Wallis Test.

In different guidelines as well as in specific population groups, cut-off values of 10^3^–10^4^ CFU/ml may be considered for the definition of UTI. In Figure 3, the Ct values of the universal probe were scattered against the bacterial load determined in culture. The results for low-level bacteriuria (10^3^–10^4^ CFU/ml) show all but one sample have Ct values of ≥30. If one would consider samples with ≥10^3^ CFU/ml as UTI and samples below 10^3^ CFU/ml as no UTI, and uses Ct 30 as a semi-quantitative breakpoint, the assay would show a sensitivity of 97%, specificity of 87%, positive predictive value of 85% and negative predictive value of 97%.

### Molecular Probes for the Identification of the Most Prevalent Uropathogens

The real-time PCR assay was, in addition to the universal 16S rDNA probe, complemented with seven genus- or species-specific probes. In this way, parallel to the differentiation between UTI and no UTI with the eubacterial 16S probe, the multiprobe assay also offered a first identification of clinically relevant uropathogens. We analyzed 279 urine specimens, of which 92 samples were considered as significant bacteriuria because of the presence of ≥10^5^ CFU/ml of a single uropathogen (Table 2. I). The Ct value derived from the universal 16S rDNA probe was used as reference point. If no other probe generated a signal within 4 Ct proximity of the universal probe, the pathogen was identified as ‘other’, being a pathogen not included in the bacterial panel. The panel of probes could identify 78% of the pathogens present in the samples with monomicrobial infections. *E. coli* was the most frequently found uropathogen (62%), and was 100% correctly identified with the specific probe. The other probes targeting *Staphylococcus* spp., *Enterococcus* spp., *Pseudomonas* spp., *Pseudomonas aeruginosa* also showed a detection rate of 100%. One sample containing a *Streptococcus* spp. was missed, resulting in a detection rate of 80%. In fourteen isolates, a polymicrobial infection was shown (Table 2. II). The assay was able to identify more than one microorganism within the same sample. As shown in Table 2 (III), in sixteen cases pathogens were found both in culture and in PCR, but were, because of the presence of low counts, considered as no UTI. Finally, 43 samples were excluded from the study due to containing contaminating skin/mixed flora as determined by conventional diagnostics. PCR analysis was performed on a subset of 11 of these samples. Results showed that in agreement with the presented data, bacterial counts could be semi-quantified and the assay used for identification. In 1 of 11 cases, the assay showed a signal for *E. coli* within 4 Ct of the universal probe, in contrast to culture data. In all remaining samples, different combinations of probes generated signals but all at high Ct values, more than 4 Ct later than the universal probe, in agreement with the culture findings of a mixed flora (data not shown).

**Table 2:** Pathogen identification of the 122 growth-positive urine specimens.

CULTURE	PCR									
Pathogen	ID probe		n			Concordance (%)				
**I. Monomicrobial UTI (n = 92)**										
*Acinetobacter baumanii*	Other^a^		1							
*beta-hemolytic Streptococcus* spp.	*Streptococcus* spp.		3/4			75%				
*Citrobacter braakii*	Other		1							
*Citrobacter freundii*	Other		1							
Coagulase-negative *Staphylococcus* spp.	*Staphylococcus* spp.		1/1			100%				
*Enterobacter cloacae*	Other		2							
*Enterococcus faecalis*	*Enterococcus* spp.		6/6			100%				
*Escherichia coli*	*Escherichia coli*		38/38			100%				
*Klebsiella ozaenae*	Other		1							
*Klebsiella pneumoniae*	Other		7							
*Proteus mirabilis*	Other	2								
*Pseudomonas aeruginosa*	*Pseudomonas aeruginosa* b		1/1			100%				
*Serratia marcescens*	Other		1							
*Staphylococcus aureus*	*Staphylococcus aureus*		1/1			100%				
*Streptococcus* spp. group B	*Streptococcus* spp.	1/1					100%			
**II. Polymicrobial UTI (n = 14)**										
*Citrobacter freundii, Enterococcus faecalis*	Other, *Enterococcus* spp., *Staphylococcus* spp.									
*Escherichia coli, Enterococcus faecalis, Candida* spp.	*Escherichia coli, Enterococcus* spp.									
*Escherichia coli, Pseudomonas aeruginosa, Enterococcus faecalis*	*Escherichia coli, Enterococcus* spp., *Pseudomonas* spp., *Pseudomonas aeruginosa*									
*Escherichia coli, Klebsiella pneumoniae*	*Escherichia coli*									
*Escherichia coli, Klebsiella pneumoniae*	*Escherichia coli*									
*Escherichia coli, Morganella morganii*	*Escherichia coli*									
*Escherichia coli, Morganella morganii*	*Other, Escherichia coli*									
*Escherichia coli, Proteus mirabilis*	*Escherichia coli*									
*Escherichia coli, Pseudomonas aeruginosa*	*Escherichia coli, Pseudomonas aeruginosa*									
*Escherichia coli,* skin flora	Other, *Staphylococcus* spp.									
*Klebsiella oxytoca, Escherichia coli*	*Escherichia coli, Streptococcus* spp.									
*Klebsiella pneumoniae, Proteus mirabilis*	Other									
*Klebsiella pneumoniae, Proteus mirabilis*	Other, *Enterococcus* spp.									
*Proteus vulgaris, Enterobacter aerogenes*	Other									
**III. Pathogens present <10^5^ CFU/ml (no UTI) n = 16**										
*Escherichia coli*	*Escherichia coli*			5/5						
*Klebsiella pneumoniae*	Other			1						
*Proteus mirabilis*	Other			4						
*Pseudomonas aeruginosa*	*Pseudomonas aeruginosa*				3/4					
*Staphylococcus aureus*	*Staphylococcus aureus*				1/1					
*Streptococcus* spp. group B	*Streptococcus* spp.			1/1						

a None of the species- or genus-specific probes generated a signal, only the universal 16S rDNA probe was positive, indicating the presence of another pathogen, not included in the bacterial panel of the real-time PCR assay.

b The isolate was identified as *P. aeruginosa*, and this was shown by positive signals from both the *Pseudomonas* spp. and the *P. aeruginosa* probe.

## Discussion

In this study, we present a novel molecular approach which utilised all capabilities of broad-spectrum real-time PCR to aid in the diagnosis of UTIs. The assay detecting the majority of uropathogens as well as a universal probe detecting eubacteria was used to distinguish urinary samples with significant, or low or no bacteriuria. Secondly, this screening assay was reinforced with rapid uropathogen identification. The assay presented in this study is based on the detection of 16S rDNA gene signatures in real-time PCR. The universal probe targeting the 16S rDNA gene was used to establish a discriminatory set point between bacteriuria and no bacteriuria. In order to prevent positive samples to be falsely considered as negative, the assay needed to have a high sensitivity and negative predictive value. A Ct value of 30 could be applied to achieve a sensitivity and NPV of 100%. However, we selected a Ct value of 25 as breakpoint because we wanted to find a proper balance between the amount of false-negative and false-positive samples. Ultimately, screening of urinary samples with our assay resulted in a sensitivity and NPV of 97% and 98%, respectively.

Thus far, another rapid approach that has been presented to distinguish bacteriuria from no bacteriuria has been the Sysmex UF-100/500/1000i urine flow cytometer. Many different studies have shown that performance characteristics could be quite different because of variable cut-off values for white blood cells (WBC) and bacteria [3], [6], [12], [13], [14], [15]. Traditionally, often significant bacteriuria is defined as the presence of ≥10^5^ CFU/ml [16], [17]. In recent years, both the American Society of Microbiology (ASM) and European urinalysis guidelines have recommended colony counts of ≥10^3^ CFU/ml of uropathogens to be reported as urinary tract infection [18]. Therefore, we also investigated the performance characteristics of the test when samples containing ≥10^3^ CFU/ml instead of ≥10^5^ CFU/ml were considered indicative of a UTI. The results showed that lowering the culture cut-off to ≥10^3^ CFU/ml could be covered by raising our semi-quantitative cut-off to Ct 30, while maintaining similar assay performance. As the assay is based on PCR, a potential disadvantage of the assay could be the detection of DNA from dead bacteria. This could be a contributing factor to the false-positive PCR results found and impact the assay’s sensitivity. In spite of this, the assay shows good performance characteristics both at cut-off of Ct 25 and Ct 30, and thus the impact of the detection of DNA cells seems limited at these Ct values.

In order to establish whether the bacterial load in urine is a result of a high load of uropathogens the second part of the assay, i.e. the identification of the pathogen, can be of great value. We saw that the absolute Ct value of the universal probe can be linked to the absolute Ct value of the specific probes. So, if the urinary sample contained a microorganism not included in the panel, the Ct value of the universal probe is that low, indicating the presence of another pathogen. From our results it became apparent that when the specific probe generated a signal within four Ct of the universal probe, we could define this as the causative agent. The current panel for this proof-of-concept study included probes for *Pseudomonas* spp., *P. aeruginosa*, *E. coli*, *Staphylococcus* spp., *S. aureus*, *Enterococcus* spp., and *Streptococcus* spp. Within our setting, the clinical relevance of the *S. aureus* probe could be discussed since it was only detected once as causative agent in our sample collection. For future studies or in other settings the assay can be adjusted to the local epidemiology and changing aetiology depending on the predominant patient group (i.e. covering pathogens found mostly in nosocomial infections or rather in outpatients/GP patients, or in populations of predominantly older or younger patients) [19], [20], [21]. Potentially interesting future targets could be *Klebsiella pneumoniae* since the prevalence was similar to *Enterococcus* spp as well as *Proteus mirabilis*. Naturally, the inclusion of additional targets will increase the costs of the assay. As shown in Table 2 III, the assay could also identify polymicrobial infections. In these cases, it was also more likely to find potential contaminants such as *Staphylococcus* spp. and *Streptococcus* spp. A report by Wu *et al.* presented a similar approach, using FISH with specific probes for the identification of uropathogens (*E. coli*, *E. faecalis* and *S. aureus*). The sensitivity of the *E. coli* specific probe was 95%, whereas the specificity of the *S. aureus* probe was 98% [22]. Lehmann *et al.* developed a real-time PCR consisting of species-specific probes for 15 pathogens and achieved a sensitivity and specificity of 90 and 87%, respectively [23]. A novel alternative to our assay is the use of Maldi-TOF analysis for identification of uropathogens. Although this approach has the advantage that it can identify a wide range of pathogens, low bacterial loads still pose a challenge for detection and no (semi- ) quantification of the bacterial load is provided [24].

Taken together, our novel approach can be of significant relevance for the rapid diagnosis of UTI because of the ability to detect both low-level and significant bacteriuria, and this combined with the identification of the uropathogen. Identification of a uropathogen is especially vital in hospital settings where there is generally a more diverse UTI aetiology. Overall, the UTI screening and identification assay showed good performance characteristics. In 78% of the monomicrobial infections, the bacterial panel could offer a tool with a correct identification rate of 99% and results were available as soon as within 4 hours. One isolate could not be identified as *Streptococcus* spp. The remaining 22% contained microorganisms not included in the bacterial panel. A limitation to this study is the inclusion of a limited number of samples (330 of which 92 showed signification bacteriuria) which is also influenced by the local epidemiology. A potential limitation of the assay is that the use of real-time PCR supplemented with molecular probes is more expensive than urine culture. Future applications concerning miniaturisation of PCR such as micro fluidic digital PCR could be used to offer rapidity, high-throughput and lower running costs, making this assay more cost-effective. Finally, in this study, screening of urinary specimens would have reduced the amount of urine specimens to be cultured with 50.5%. In summary, the assay could be of great value to improve the diagnosis and therapy of UTIs, especially in hospital settings.
